# Stereotactic heart ablative radiotherapy (SHARP): a prospective multicentric phase II trial

**DOI:** 10.1007/s00066-026-02504-5

**Published:** 2026-01-30

**Authors:** Aurélie Gaasch, Sebastian N. Marschner, Philipp Hoegen-Saßmannshausen, Elisabetta Sandrini, Juliane Hörner-Rieber, Nicolaus Andratschke, Panagiotis Balermpas, Luca Boldrini, Angela Romano, Michael Reiner, Maximilian Niyazi, Lars Lindner, Ludwig Weckbach, Nicola Fink, Christian Hagl, Claus Belka, Stefanie Corradini

**Affiliations:** 1 Department of Radiation Oncology, LMU University Hospital, Munich, Germany; 2 German Cancer Consortium (DKTK) Partner Site Munich, German Cancer Research Center (DKFZ), Heidelberg, Australia; 3 Department of Radiation Oncology, University Hospital Heidelberg, Heidelberg, Germany; 4 Department of Radiation Oncology, University Hospital Düsseldorf, Düsseldorf, Germany; 5 University Hospital Zurich, Department of Radiation Oncology, University of Zurich, Zurich, Switzerland; 6 Fondazione Policlinico Universitario “A. Gemelli” IRCCS, Rome, Italy; 7 Department of Radiation Oncology, University Hospital Tübingen, Tübingen, Germany; 8 Department of Hematology and Oncology, LMU University Hospital, Munich, Germany; 9 Department of Cardiology, LMU University Hospital, Munich, Germany; 10 Department of Radiology Munich, LMU University Hospital, Munich, Germany; 11 Department of Cardiac surgery, LMU University Hospital, Munich, Germany; 12 Bavarian Cancer Research Center (BZKF), Munich, Germany

**Keywords:** SBRT, Online adaptive, MR-guidance, Cardiac, MRgSBRT

## Abstract

**Background:**

Cardiac tumors are exceedingly rare with metastatic involvement representing the most frequent form of malignancy within the heart. In patients presenting with inoperable primary or recurrent malignant cardiac sarcomas or cardiac/epicardial/pericardial metastases, stereotactic body radiotherapy (SBRT) serves as an alternative local treatment to surgery or may, in some cases, constitute the sole viable local treatment modality. To date, there are only case reports or small retrospective studies assessing SBRT dose and toxicity for cardiac SBRT. The goal of this prospective multicentric observational study is to systematically evaluate the feasibility, toxicity and outcome of magnetic resonance-guided stereotactic body radiation therapy (MRgSBRT) in the management of primary and secondary cardiac malignancies.

**Methods:**

The treatment is performed using MR-guided SBRT in five fractions on non-consecutive days (6–8 Gy per fraction prescribed to the 80% isodose). Four study centers participate in this prospective phase II trial (Heidelberg, Munich, Rome, Zurich). Eligible patients who consent to participate in the study will undergo treatment as indicated and approved by an interdisciplinary tumor board. Primary objective is to assess the feasibility and safety of online adaptive MRgSBRT; secondary endpoints are local control and survival outcome, acute and late toxicity, patient-reported outcome as well as technical feasibility of treatment.

**Discussion:**

The findings from this study may serve as a foundation for the future integration of cardiac SBRT into clinical practice guidelines for the management of cardiac malignancies.

## Background

Cardiac tumors are generally very rare [1]. The most frequent malignant tumors within the heart are metastases, mainly from pleural mesothelioma, malignant melanoma, lung cancer, breast cancer, and ovarian carcinomas [2]. Cardiac metastatic lesions can occur throughout the heart but are mainly located in the pericardium (69%), followed by the epicardium and myocardium [2]. Primary cardiac tumors are even less frequent and represent a heterogeneous group of neoplasms with different clinical courses and histologies. They are benign in about 75% of cases and malignant in 25%, with atrial myxoma being the most common primary tumor of the heart. Primary malignant tumors are frequently sarcomas (65%), with angiosarcomas being the most common malignant tumor and the secondarily most common being lymphomas (27%) [3]. Generally, cardiac sarcomas can occur in any part of the heart, but angiosarcomas are typically found in the right atrium [4, 5], while fibrosarcomas and undifferentiated sarcomas are predominantly located in the left atrium [5, 6]. Primary malignant cardiac tumors have an incidence of about 34 cases per 100 million people (incidences range from 0.001% to 0.28%), with a prevalence of primary cardiac sarcomas of about 0.0017%, while primary cardiac angiosarcomas have an incidence of 0.001%–0.03% at autopsy [7]. Patients with malignant cardiac tumors are more frequently female (54.1%) and have a median age at diagnosis of 50 years [3].

### Symptoms

The symptoms associated with cardiac sarcomas and cardiac metastases are manifold and depend on the location of the tumor. They range from heart-specific symptoms such as cardiac arrhythmias, congestive heart failure, syncope, pericardial effusions, and blood clots to general symptoms such as chest pain or shortness of breath and accompanying neoplastic symptoms like fever, weight loss, and malaise [5]. Changes in the echocardiogram (ECG) are common in cardiac metastases, but many cardiac neoplasms are not detected during the lifetime and are only diagnosed postmortem, as 90% of cardiac metastases are clinically silent [2, 8].

### Diagnostics

Transthoracic (TTE) and transesophageal (TEE) echocardiography are useful diagnostic tools due to their high sensitivity. Furthermore, imaging using computed tomography (CT) scans or cardiac-gated magnetic resonance imaging (MRI) are also frequently used for detection and evaluation of cardiac lesions [5, 7]. Most patients with primary heart tumors are diagnosed with a tissue sample (96.8%) [3].

### Standard of care

**Fig. 1:**
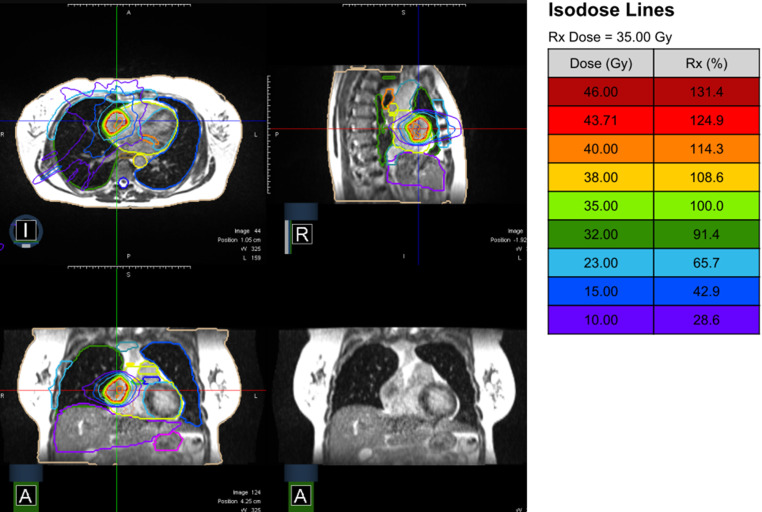
Exemplary SBRT of cardiac sarcoma treated with MR-guided RT with 5 x 6 Gy (dosed on the 80% isodose). Isodose lines (*right*) showing the dose (Gy) and percentage (%) of the prescribed dose.

Due to the heterogeneity and rarity of primary and recurrent cardiac sarcomas and cardiac metastases, there are no uniform treatment guidelines to date. Treatment options include radical surgery (if feasible) followed by radiotherapy and/or chemotherapy with anthracyclines, ifosfamide, or taxanes (primary cardiac sarcomas) or tumor-directed systemic therapies in cases of metastases. Cardiac transplantation represents an emerging strategy for patients with isolated inoperable cardiac involvement [5]. Over the past years, stereotactic body radiotherapy (SBRT) has become a new alternative treatment option for functionally inoperable cardiac and pericardiac malignancies [9–12]. Real-time magnetic resonance imaging (MRI)-guided radiotherapy (MRgRT) represents one of the most innovative applications of current image-guided radiation therapy (IGRT) [13]. Novel hybrid devices combine magnetic resonance imaging and a linear accelerator in a single device. The revolutionary concept of MRgRT creates new perspectives in radiotherapy (RT) based on the use of high-quality image guidance. The MR images acquired immediately before and online during the treatment enable daily plan adaptation strategies to improve targeting accuracy while avoiding critical structures. The superior soft tissue contrast compared with cone-beam computed tomography (CBCT)-based treatment and the possibility of daily plan adaptation promise higher treatment precision as well as better target volume coverage and normal tissue sparing. The present study tests the feasibility of imaging and treatment using MR-guided SBRT. Especially in the case of cardiac malignancies, MRgRT enables direct visualization of the tumor during treatment. Respiratory-gated SBRT allows for reduced planning target volume margins, which may help to minimize treatment-related toxicity. An example is provided in Fig. 1.

### Outcome

The overall survival outcome in primary malignant cardiac sarcomas is poor and ranges from a median of 6 to 12 months. One study analyzing only primary cardiac sarcomas between 1973 to 2015 found survival rates of 40.7% at 1 year, 15.6% at 3 years, and 9.8% at 5 years [14]. In a recent Surveillance, Epidemiology, and End Results (SEER) analysis of 551 primary malignant cardiac tumors treated between 1973 and 2011, the median overall survival rates at 1, 3, and 5 years were 46%, 22%, and 17%, respectively. Overall, an improvement in outcomes was observed over time, with 1‑, 3‑, and 5‑year survival rates of 32%, 17%, and 14% for patients treated between 1973 and 1989 and of 50%, 24%, and 19% for patients treated from 2000 to 2011, respectively (*p* = 0.009) [3]. In 2025, insights from two decades of management of cardiac sarcomas from the LMU Munich (2002–2024) were published, showing a median overall survival of 37.5 months [15]. This favorable outcome was attributed to the high proportion of patients receiving multimodal treatment at this institution during the past 20 years.

While survival in patients with cardiac and pericardial metastases depends mainly on the primary cancer entity, the presence or absence of disseminated metastatic disease, and whether the heart is the only site of presentation (oligometastatic), the treatment options can range from palliative to curative therapy. So far, data on the outcomes of SBRT are scarce, and only case reports or smaller case series have been reported. Nevertheless, favorable outcomes with good local response rates of up to 75% after using 3 × 8–13 Gy (prescribed to the 70%–80% isodose [ID]), 5 × 6 Gy (80% ID), or 1 × 20 Gy (88% ID) SBRT have been reported [9–12].

## Methods

### Study design and objective

This study was designed as a prospective multicenter observational phase II trial evaluating the feasibility and safety of online adaptive MR-guided SBRT in cardiac/epicardial/paracardial lesions. Participating study centers comprise the radiation oncology departments of the University Hospital Heidelberg, the University Hospital Munich, the Agostino Gemelli University Hospital Rome, and the University Hospital Zurich. The primary endpoint is a combined endpoint for which occurrence of any of the following events is considered an event:

- any acute toxicity > CTCAE grade 2 within 3 months after RT initiation (Common Terminology Criteria for Adverse Events CTCAE v5).
- treatment discontinuation, whereby a connection with the treatment must exist.
- mortality within 3 months after RT start (related to treatment and/or disease).

The secondary endpoint comprises the oncologic outcome, including local control and survival as well as acute and late toxicity. Furthermore, patient-reported outcome measures as well as the technical feasibility of treatment (intrafractional image quality and gating, frequency of plan adaptation, motion analysis, duty cycle of respiratory gating, physical assessment of treatment plans) represent further secondary study outcomes.

The study was set to start recruitment in 2022, with the aim of recruiting 10–15 patients per year due to the rare incidence. The inclusion period was planned to last 2 years but was then extended until the fourth quarter of 2025 by an amendment in 2024.

### Patient population

Inclusion criteria according to the protocol are:

- ability to comply with the study protocol and provide informed consent
- age ≥ 18 years
- inoperable primary or recurrent malignant cardiac sarcomas or cardiac/pericardial metastases or patients who refuse surgery
- ECOG performance status 0–2

Exclusion criteria according to the protocol are:

- patients without legal capacity or who are unable to understand the nature, significance, and consequences of the study
- simultaneous participation in other interventional trials which could interfere with this trial
- pregnancy
- contraindication to MRI

### Statistical analysis

A per-protocol analysis will be performed for all patients treated in the study. All results will be interpreted descriptively. Kaplan–Meier curves will be used to assess local control and overall survival rates. Adverse events will be reported with severity grades according to CTCAE. All data will be analyzed using SPSS Statistics as well as Excel (newest versions available; IBM Corp., Armonk, NY, USA and Microsoft, Redmond, WA, USA, respectively). Data management is performed using RedCap hosted at the IBE Munich (Institut für medizinische Informationsverarbeitung, Biometrie und Epidemiologie University Hospital LMU Munich).

### Treatment planning: target delineation, planning objectives, and mRgSBRT delivery

Radiotherapy planning follows the standard procedures of MR-guided RT of each institution and includes MRI simulation using a dedicated 0.35 T hybrid MR-Linac (ViewRay, Oakwood Village, OH, USA). Multiple scans using breath-hold techniques are usually acquired to obtain a reproducible and stable breath-hold and define the position best tolerated by the patient. Thereafter, a planning CT using the same patient positioning and the same breath-hold is performed for electron density information. These two imaging sets are then fused, and target delineation is performed. The target volume (gross tumor volume, GTV) and the organs at risk (OAR; heart, heart valves, heart minus PTV, aorta, spinal cord, spinal canal, lungs, trachea, esophagus, etc.) are delineated. The GTV is isotropically expanded by 3(–5) mm to define the planning target volume (PTV). The dose range is 5 × 6–8 Gy to the 80% isodose (non-consecutive days), depending on the tumor size and localization, and might be individualized on a case-by-case basis. During dose delivery, a uniform 3–5 mm margin is used for real-time gating, allowing a pixel excursion tolerance of 3%–8%. Gating is preferably performed on the GTV or, alternatively, by using a surrogate (Table 1).

**Table 1:** Dose constraints for treatment planning for 5 fractions

Structure	Volume of dose	Dmax
PTV	> 95–99% of PD	125% of PD (aimed Dmax) ± 5%
Spinal cord	< 0.35 cc at 23 Gy	30 Gy
Heart valves (aortic, pulmonary, mitral, tricuspid) excluding PTV	< 0.5 cc at 23 Gy**soft constraint	38 Gy**soft constraint
	< 1 cc at 36 Gy	40 Gy
Left ventricle excluding PTV	< 0.5 cc at 35 Gy**soft constraint	38 Gy**soft constraint
	< 1 cc at 36 Gy	40 Gy
Heart excluding PTV	< 15 cc at 32 Gy	38 Gy**soft constraint
		40 Gy
Great vessels	< 10 cc at 47 Gy	53 Gy
Trachea	< 4 cc at 16.5 Gy	40 Gy
Bronchus	< 0.5 cc at 21 Gy	33 Gy
Esophagus	< 1 cc at 19.5 Gy	35 Gy
Bowel	< 0.5 cc at 33 Gy	–
Stomach	< 0.5 cc at 33 Gy	–

*Dmax *maximum dose, *PD *prescription dose, *PTV *planning target volume

Eligible patients who consent to participate in the study will undergo treatment as indicated and approved by an interdisciplinary tumor board.

### Follow-up

Overall documentation at baseline and follow-up is shown in Table 2. Serial assessments before and every 3 months after radiation treatment will be performed.

**Table 2:** Workflow of the SHARP-study

Workflow	Baseline visit	End of RT	Follow-up			
	T0	T1	T2	T3	T4	T5
	2 weeks before RT (day −13 until day 0)	End of RT	3 months after RT (±10 days)	6 months after RT (±10 days)	9 months after RT (±10 days)	12 months after RT (±10 days)
Check of in-/exclusion criteria	x	–	–	–	–	–
Patient information, informed consent	x	–	–	–	–	–
Registration	x	–	–	–	–	–
Documentation of demographic data, medical history, family history, and baseline symptoms	x	–	–	–	–	–
Documentation of feasibility of MR-guided radiotherapy	–	x	–	–	–	–
Clinical examination (incl. NYHA score, weight/height, and vital signs)	x	x	x	x	x	x
ECOG performance Status	x	x	x	x	x	x
laboratory Examination (optional)	(x)	–	(x)	(x)	(x)	(x)
Documentation of medication (including anticoagulation and systemic therapy)	x	x	x	x	x	x
Documentation of toxicities and adverse effects (CTCAE)	–	x	x	x	x	x
QoL assessment (QLQ-C30, KCCQ; optional)	(x)	(x)	(x)	(x)	(x)	(x)
Diagnostic imaging (CT and/or MRI)	x	–	x	x	x	x
Cardiac assessment/function (echocardiography, left ventricular ejection fraction, alterations in ECG or MRI; optional)	(x)	–	(x)	(x)	(x)	(x)
Documentation of local control and survival status	–	–	x	x	x	x

*CTCAE* Common Terminology Criteria for Adverse Events, *QLQ* Quality of Life Questionnaire, *KCCQ* Kansas City Cardiomyopathy Questionnaire, *ECG* echocardiography

At baseline, documentation includes obtaining informed consent, recording demographic data and medical history, and conducting a clinical examination that encompasses the New York Heart Association (NYHA) classification as well as measurements of body weight and height. The ECOG performance status is assessed, along with documentation of current medication, particularly anticoagulation therapy. If possible, health-related quality of life (HR-QoL) will be assessed by the European Organization for Research and Treatment of Cancer (EORTC) Quality of Life Core Questionnaire for Cancer Patients (QLQ C30), the Kansas City Cardiomyopathy Questionnaire (KCCQ), and the New York Heart Association (NYHA) functional classification to classify the extent of heart failure. Diagnostic imaging via CT and/or MRI is performed. If possible, cardiac function is assessed via transthoracic echocardiography, including detailed evaluation of left ventricular parameters such as Simpson biplane measurements, 3D left ventricular ejection fraction, 3D systolic and diastolic volumes, global longitudinal strain, and diastolic function. Right ventricular parameters are also measured, including TAPSE (tricuspid annular plane systolic excursion), RV diameters, RV systolic and diastolic volumes, 3D RV function, and global longitudinal strain. Both left and right atrial dimensions are documented, along with an evaluation of the heart valves. A laboratory examination (creatinine, troponin, creatine kinase [CK], CK-MB, NT-proBNP) can be additionally performed.

At the end of radiation treatment, the feasibility of the MRgSBRT treatment is assessed, as are treatment discontinuation, interruptions, and surpassed dose constraints. Clinical examination is performed, and medication changes and acute toxicity during radiation treatment are registered. If possible, the quality of life assessment is repeated.

At subsequent study visits (every 3 months), documentation continues with clinical examinations that include the NYHA score, body weight, and height measurements. The ECOG performance status and medication records are updated. If possible, cardiac function is again assessed using transthoracic echocardiography with the same detailed parameters for the left and right ventricles, atria, and valve evaluations as at baseline. If possible, laboratory examinations can be performed using the same parameters as for baseline. Quality of life assessments using the QLQ-C30 and KCCQ are repeated. Additionally, documentation at these visits includes recording acute and late toxicities according to the Common Terminology Criteria for Adverse Events (CTCAE) v5.0 as well as survival status. Regular imaging with CT and/or MRI will be performed for the evaluation of local disease control, as defined in the institutional standard operating procedures (SOPs).

### Study coordination and registration

The study is coordinated by the Department of Radiation Oncology at the LMU Munich, with the principal investigator based at the LMU. The LMU Munich oversees trial management, database administration, and reporting. Patient recruitment and treatment are carried out at the radiation oncology departments of the University Hospitals of Heidelberg, Munich, Zurich, and the Agostino Gemelli University Hospital in Rome.

The study is conducted in accordance with the Declaration of Helsinki and has received ethics approval from the Ethics Committee of the Medical Faculty of the LMU Munich (no. 21-0696) and is registered at the German Study Registry (no. DRKS00027108). The study is also registered as an ARO study (study number 2024-09). Informed consent is obtained from all participants.

## Outlook

The case reports available to date suggest that SBRT may be an effective non-invasive treatment option, particularly for inoperable or systemically refractory cardiac tumors [16, 17]. The combination of real-time imaging and adaptive plan adjustment in MRgSBRT opens up new therapeutic options in an anatomically and functionally highly sensitive area; MRgSBRT also offers the option of daily plan adjustment (online adaptive radiotherapy), which can increase dose conformity and safety, thereby offering a low-toxicity treatment for patients with currently limited treatment options and a consequently poor oncologic prognosis [18]. The SHARP prospective multicenter international study will provide initial clinical data from a larger cohort on the use of MR-guided stereotactic radiotherapy in patients with primary or secondary cardiac tumors.

This prospective phase II study involves four university hospitals in Germany, Switzerland, and Italy. Patients with rare cardiac tumors and metastases should always be treated at highly specialized centers/university hospitals, in which experts from radiology, oncology (if possible, sarcoma centers), cardiology, cardiac surgery, and radiotherapy collaborate closely. Only through this comprehensive approach can all therapeutic options be thoroughly evaluated, thereby ensuring an optimal individualized treatment strategy for each patient.

If showing promising oncologic outcomes as well as no higher toxicity, this study could be the first step toward integrating cardiac SBRT into treatment guidelines for cardiac malignancies and open up the landscape for further prospective studies to refine and optimize this novel treatment approach.

## Data Availability

Not applicable (no data in the manuscript). Data management is performed using RedCap hosted at the IBE Munich (Institut für medizinische Informationsverarbeitung, Biometrie und Epidemiologie University Hospital LMU Munich).

## Abbreviations

CBCT: Cone-beam computed tomography
CTCAE: Common Terminology Criteria for Adverse Events
GTV: Gross tumor volume
IGRT: Image-guided radiation therapy
MRgSBRT: Magnetic resonance-guided SBRT
PTV: Planning target volume
SBRT: Stereotactic body radiotherapy
TTE/TEE: Transthoracic/transesophageal echocardiography
